# Supplementary material for: Integrated Approach for Synthetic Cathinone Drug Prioritization and Risk Assessment: In Silico Approach and Sub-Chronic Studies in Daphnia magna and Tetrahymena thermophila
Source: Molecules. 2023 Mar 23;28(7):2899. doi: 10.3390/molecules28072899 (PMC10096003; doi:10.3390/molecules28072899)
Supplement: Supplementary file 1 [file molecules-28-02899-s001.zip › molecules-2281761-supplementary.pdf]

Supplementary Material

Integrated Approach for Synthetic Cathinone Drug Prioritization and Risk Assessment: In Silico Approach and Sub-Chronic Studies in Daphnia magna and Tetrahymena thermophila

Ariana Pérez-Pereira [1,2], Ana Rita Carvalho [1], João Soares Carrola [2,3], Maria Elizabeth Tiritan [1,4,5,*] and Cláudia Ribeiro [1,*]

[1] TOXRUN—Toxicology Research Unit, University Institute of Health Sciences, IUCS-CESPU, CRL, 4585-116 Gandra, Portugal

[2] Department of Biology and Environment, University of Trás-os-Montes and Alto Douro (UTAD), CITAB, 5000-801 Vila Real, Portugal

[3] Inov4Agro—Institute for Innovation, Capacity Building and Sustainability of Agri-Food Production, 5000-801 Vila Real, Portugal

[4] Interdisciplinary Center of Marine and Environmental Research (CIIMAR), University of Porto, Edifício do Terminal de Cruzeiros do Porto de Leixões, 4450-208 Matosinhos, Portugal

[5] Laboratory of Organic and Pharmaceutical Chemistry, Department of Chemical Sciences, Faculty of Pharmacy, University of Porto, 4050-313 Porto, Portugal

* Correspondence: elizabeth.tiritan@iucs.cespu.pt (M.E.T.); claudia.ribeiro@iucs.cespu.pt (C.R.)

1. RESULTS AND DISCUSSIONS

1.1. In silico studies

Table S1 presents the Chemical Abstracts Service (CAS) registry numbers, the Simplified Molecular Input Line Entry System (SMILES) notations and the polar surface area (PSA) value for each of the 44 SC obtained through the PubChem online platform. The 44 SC were divided into the following seven groups considering PSA values (ranged from 20.3 to 57.3 Å^2): 20.3 Å^2 for

(*S*)-MTFP ((*S*)-metamfepramone or *N*,*N*-dimethylcathinone), MPP, 4-MPBP (4-methyl-α-pyrrolizinobutyrophenone), NPP (naphthylpyrovalerone or naphyrone), α-PVP (α-pyrrolidinovalerophenone), 5-PPDI (indanyl-α-pyrrolidinobutiophenone), 4-BrPVP (4-bromo-α-pyrrolidinopentiophenone), 4-FPHP (4-fluoro-α-pyrrolidinohexanophenone), DMP (dimethylpentedrone), α-PHP (α-pyrrolidinohexanophenone) and α-PIHP (α-pyrrolidinoisohexanophenone); 29.1 Å^2 for 3,4-DMMC, 3-MMC, BPD, 4-MMC, EPH, EPP (ethcathinone), TBCP (bupropion or amfebutamone), 4-MEC (4-methylethcathinone), 4-FMC (4-fluoromethcathinone or flephedrone), 3-FMC (3-fluoromethcathinone or 3-flephedrone), 4-MPD (4-methylpentedrone), α-BHP (α-butylaminohexanophenone), 4-BMC (4-bromomethcathinone or brephedrone), MPH (hexedrone), 2,4-DMEC (2,4-dimethylethcathinone), 2,4-DMMC (2,4-dimethylmethcathinone or 2-methylmephedrone), BMAPN (2-(methylamino)-1-(naphthalen-2-yl)propan-1-one), α-PPP (α-propyloaminopentiophenone or *N*-propylpentedrone) and PTD; 29.5 Å^2 for 4-MeO-α-PHPP (4-methoxy-α-pyrrolidinoheptanophenone), 4-MeO-α-POP (4-methoxy-α-pyrrolidinooctanophenone) and 4-MeO-α-PVP (4-methoxy-α-pyrrolidinovalerophenone); 38.8 Å^2 for MDPV, MDPBP (3,4-methylenedioxy-α-pyrrolidinobutyrophenone) and 3,4-DMPVP (3,4-dimethoxy-α-pyrrolidinopentiophenone); 43.1 Å^2 for CATH; 47.6 Å^2 for BTL, bk-MDEA (ethylone), bk-EBDB (eutylone), bk-MAP, bk-EBDP (*N*-ethylpentylone) and bk-MBDP (pentylone); and 57.3 Å^2 for MTP (thiothinone).

Table S1: Acronym, International Union of Pure and Applied Chemistry (IUPAC) name, Chemical Abstracts Service (CAS) number, chemical structure, Simplified Molecular Input Line Entry System (SMILES) notation and polar surface area (PSA) value of 44 SC for *in silico* studies.

SC (IUPAC name)	Acronym	CAS number	Structure	SMILES	Topological polar surface area (PSA; Å²)
**Cathinone or norephedrone** ((2*S*)-2-(Amino)-1-(phenyl)propan-1-one)	CATH	71031-15-7	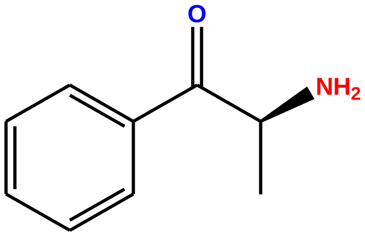	CC(C(=O)C1=CC=CC=C1)N	43.1
**Butylone** (1-(1,3-Benzodioxol-5-yl)-2-(methylamino)butan-1-one)	BTL	802575-11-7	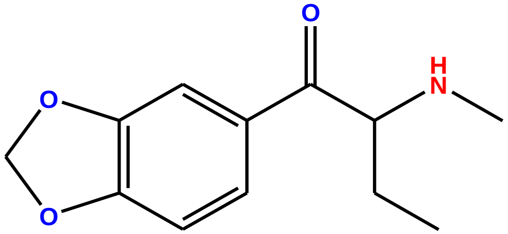	CCC(C(=O)C1=CC2=C(C=C1)OCO2)NC	47.6
**3,4-Methylenedioxypyrovalerone** (1-(1,3-Benzodioxol-5-yl)-2-(1-pyrrolidinyl)pentan-1-one)	MDPV	687603-66-3	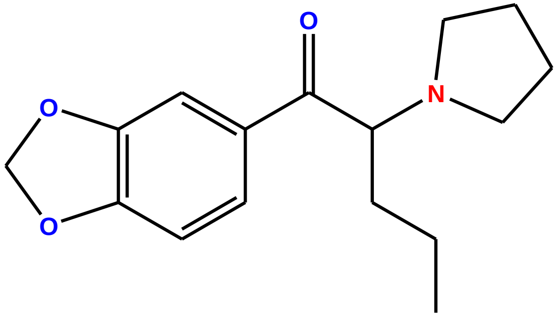	CCCC(C(=O)C1=CC2=C(C=C1)OCO2)N3CCCC3	38.8

**3,4-Dimethylmethcathinone**<br>(1-(3,4-Dimethylphenyl)-2-(methylamino)propan-1-one)	3,4-DMMC	1082110-00-6	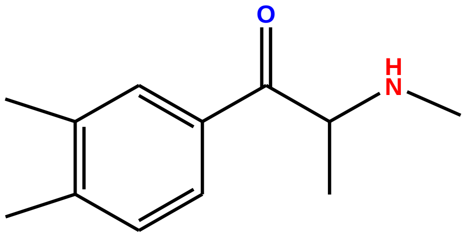	CC1=C(C=C(C=C1)C(=O)C(C)N C)C	29.1
**3-Methylmethcathinone**<br>(2-(Methylamino)-1-(3-methylphenyl)propan-1-one)	3-MMC	1246816-62-5<br>or<br>1246911-86-3	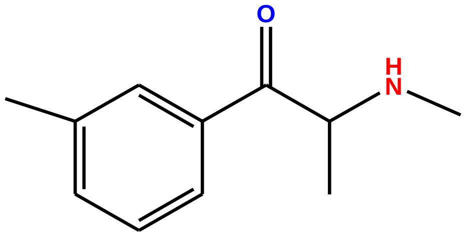	CC1=CC(=CC=C1)C(=O)C(C)NC	29.1
**Buphedrone**<br>(2-(Methylamino)-1-(phenyl)butan-1-one)	BPD	408332-79-6	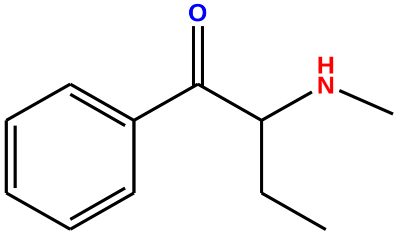	CCC(C(=O)C1=CC=CC=C1)NC	29.1
**Ethylone**<br>(1-(1,3-Benzodioxol-5-yl)-2-(ethylamino)propan-1-one)	bk-MDEA	1112937-64-0	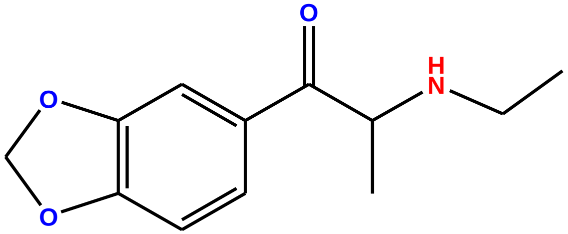	CCNC(C)C(=O)C1=CC2=C(C=C 1)OCO2	47.6

**Eutylone**<br>(1-(1,3-Benzodioxol-5-yl)-2-(ethylamino)butan-1-one)	bk-EBDB	802855-66-9	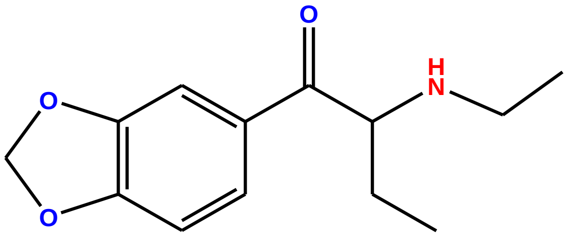	CCC(C(=O)C1=CC2=C(C=C1)OCO2)NCC	47.6
**Mephedrone or 4-methylmethcathinone**<br>(2-(Methylamino)-1-(4-methylphenyl)propan-1-one)	4-MMC	1189805-46-6	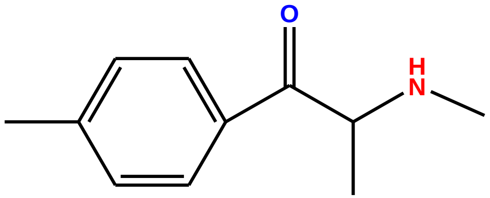	CC1=CC=C(C=C1)C(=O)C(C)NC	29.1
**Methcathinone or ephedrone**<br>(2-(Methylamino)-1-(phenyl)propan-1-one)	EPH	5650-44-2	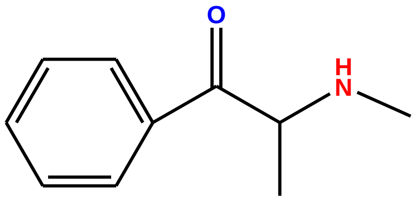	CC(C(=O)C1=CC=CC=C1)NC	29.1
**Methylone**<br>(1-(1,3-Benzodioxol-5-yl)-2-(methylamino)propan-1-one)	bk-MAP	186028-79-5	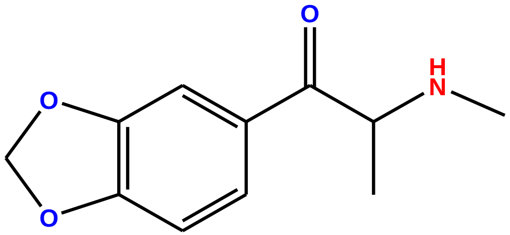	CC(C(=O)C1=CC2=C(C=C1)OCO2)NC	47.6

***N*-Ethylpentylone**<br>(1-(1,3-Benzodioxol-5-yl)-2-(ethylamino)pentan-1-one)	bk-EBDP	727641-67-0	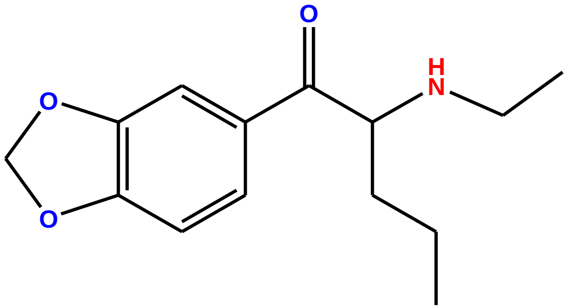	CCCC(C(=O)C1=CC2=C(C=C1)OCO2)NCC	47.6
**Pentylone**<br>(1-(1,3-Benzodioxol-5-yl)-2-(methylamino)pentan-1-one)	bk-MBDP	698963-77-8	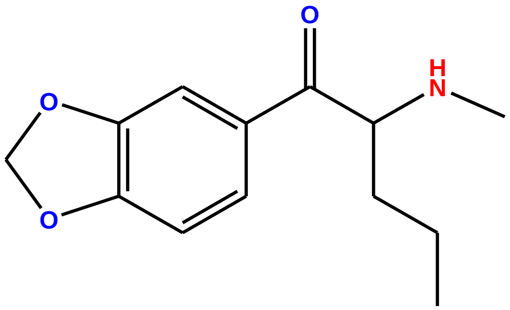	CCCC(C(=O)C1=CC2=C(C=C1)OCO2)NC	47.6
**(*S*)-Metamfepramone or *N*,*N*-dimethylcathinone**<br>((2*S*)-2-Dimethylamino)-1-(phenyl)propan-1-one)	(*S*)-MTFP	35026-77-8	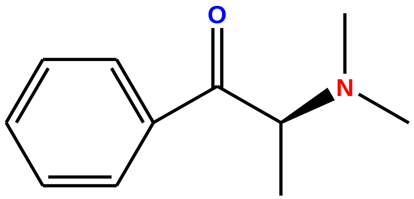	CC(C(=O)C1=CC=CC=C1)N(C)C	20.3
**Ethcathinone**<br>(2-(Ethylamino)-1-(phenyl)propan-1-one)	EPP	18259-37-5	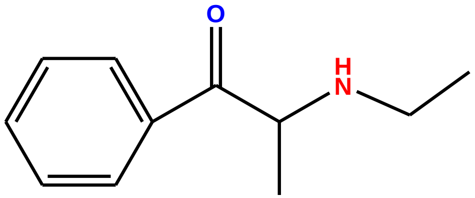	CCNC(C)C(=O)C1=CC=CC=C1	29.1

**Pyrovalerone**<br>(1-(4-Methylphenyl)-2-(pyrrolidin-1-yl)pentan-1-one)	MPP	3563-49-3	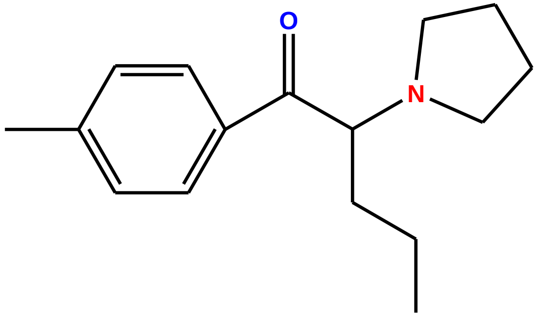	CCCC(C(=O)C1=CC=C(C=C1)C)N2CCCC2	20.3
**4-Methyl-α-pyrrolizinobutyrophenone**<br>(1-(4-Methylphenyl)-2-(pyrrolidin-1-yl)butan-1-one)	4-MPBP	732180-91-5	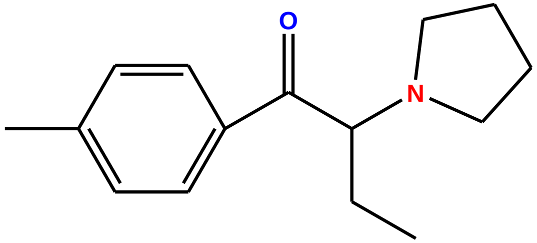	CCC(C(=O)C1=CC=C(C=C1)C)N2CCCC2	20.3
**Naphthylpyrovalerone or naphyrone**<br>(1-(Naphthalen-2-yl)-2-(pyrrolidin-1-yl)pentan-1-one)	NPP	850352-53-3	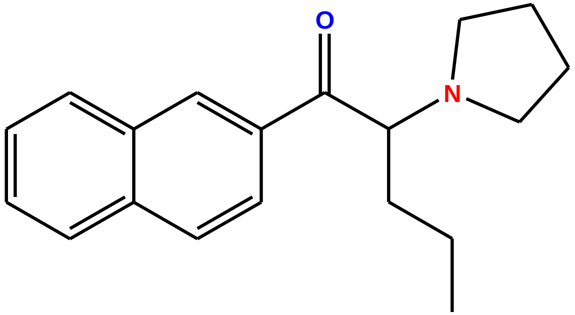	CCCC(C(=O)C1=CC2=CC=CC=C2C=C1)N3CCCC3	20.3

**α-Pyrrolidinovalerophenone** (1-(Phenyl)-2-(pyrrolidin-1-yl)pentan-1-one)	α-PVP	14530-33-7	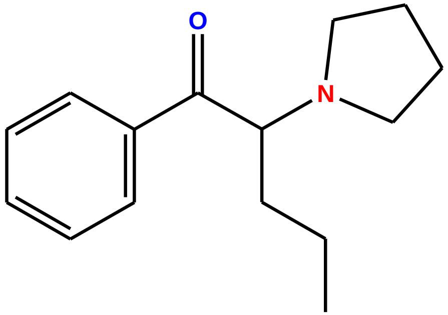	CCCC(C(=O)C1=CC=CC=C1)N2 CCCC2	20.3
**3,4-Methylenedioxy-α-pyrrolidinobutyrophenone** (1-(1,3-Benzodioxol-5-yl)-2-(pyrrolidin-1-yl)butan-1-one)	MDPBP	784985-33-7	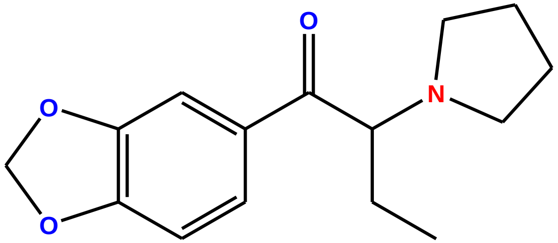	CCC(C(=O)C1=CC2=C(C=C1)O CO2)N3CCCC3	38.8
**Bupropion or amfebutamone** (2-(Tert-butylamino)-1-(3-chlorophenyl)propan-1-one)	TBCP	34911-55-2 or 34841-39-9	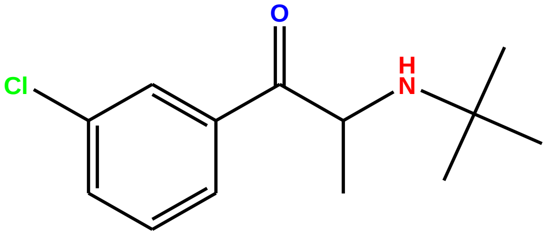	CC(C(=O)C1=CC(=CC=C1)Cl)N C(C)(C)C	29.1
**4-Methylethcathinone** (2-(Ethylamino)-1-(4-methylphenyl)propan-1-one)	4-MEC	1225617-18-4	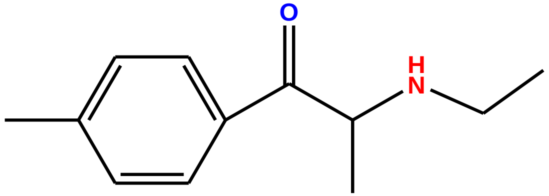	CCNC(C)C(=O)C1=CC=C(C=C1) C	29.1

**4-Fluoromethcathinone or flephedrone** (1-(4-Fluorophenyl)-2-(methylamino)propan-1-one)	4-FMC	447-40-5	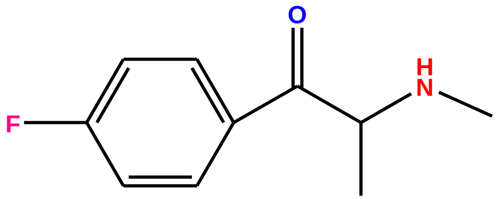	CC(C(=O)C1=CC=C(C=C1)F)NC	29.1
**3-Fluoromethcathinone or 3-flephedrone** (1-(3-Fluorophenyl)-2-(methylamino)propan-1-one)	3-FMC	1049677-77-1	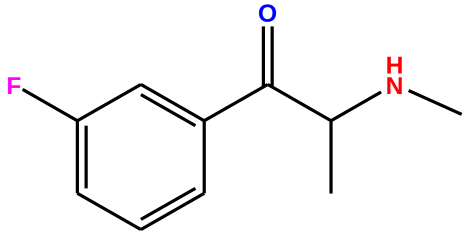	CC(C(=O)C1=CC(=CC=C1)F)NC	29.1
**4-Methylpentedrone** (2-(Methylamino)-1-(4-methylphenyl)pentan-1-one)	4-MPD	1373918-61-6	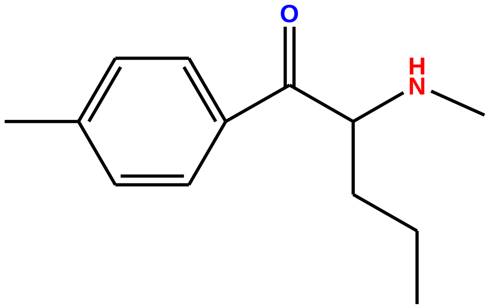	CCCC(C(=O)C1=CC=C(C=C1)C)NC	29.1
**Thiothinone** (2-(Methylamino)-1-(thiophen-2-yl)propan-1-one)	MTP	24065-17-6	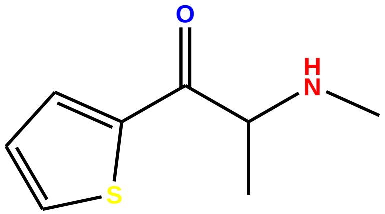	CC(C(=O)C1=CC=CS1)NC	57.3

**Indanyl-α-pyrrolidinobutiophenone** (1-(2,3-Dihydro-1*H*-inden-5-yl)-2-(pyrrolidin-1-yl)butan-1-one)	5-PPDI	2304915-05-5	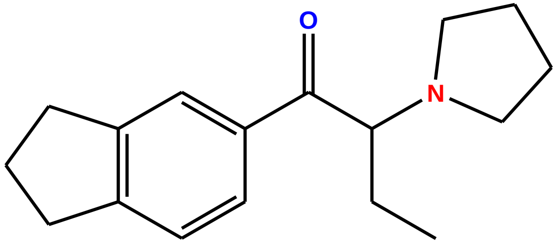	CCC(C(=O)C1=CC2=C(CCC2)C=C1)N3CCCC3	20.3
***α*-Butylaminohexanophenone** (2-(Butylamino)-1-(phenyl)hexan-1-one)	*α*-BHP	802576-87-0	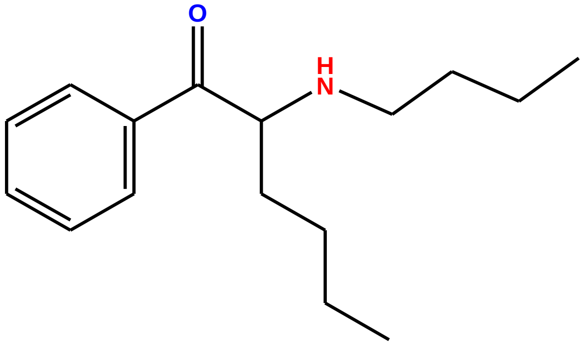	CCCCC(C(=O)C1=CC=CC=C1)NCCCC	29.1
**4-Bromomethcathinone or brephedrone** (1-(4-Bromophenyl)-2-(methylamino)propan-1-one)	4-BMC	486459-03-4	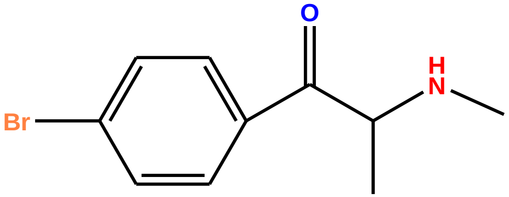	CC(C(=O)C1=CC=C(C=C1)Br)NC	29.1

**Hexedrone**<br>(2-(Methylamino)-1-(phenyl)hexan-1-one)	MPH	2169446-41-5	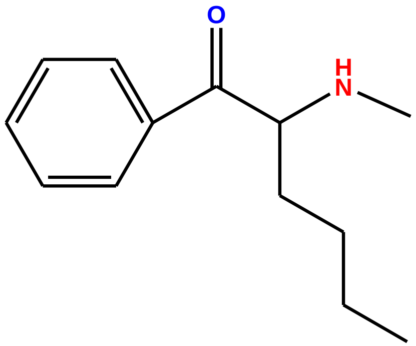 	CCCCC(C(=O)C1=CC=CC=C1)N<br>C	29.1
**2,4-Dimethylethcathinone**<br>(1-(2,4-Dimethylphenyl)-2-<br>(ethylamino)propan-1-one)	2,4-DMEC	1225913-88-1	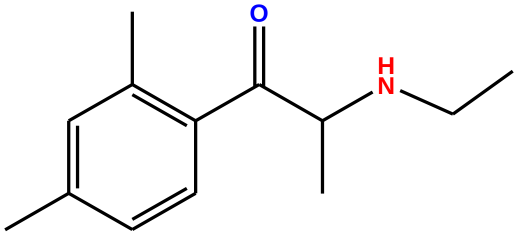 	CCNC(C)C(=O)C1=C(C=C(C=C1<br>)C)C	29.1
**2,4-Dimethylmethcathinone or 2-methylmephedrone**<br>(1-(2,4-Dimethylphenyl)-2-<br>(methylamino)propan-1-one)	2,4-DMMC	1225623-63-1	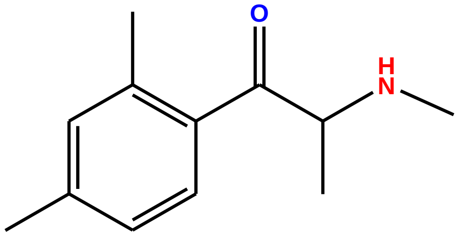 	CC1=CC(=C(C=C1)C(=O)C(C)N<br>C)C	29.1

**3,4-Dimethoxy-α-pyrrolidinopentiophenone** (1-(3,4-Dimethoxyphenyl)-2-(pyrrolidin-1-yl)pentan-1-one)	3,4-DMPVP	850442-84-1	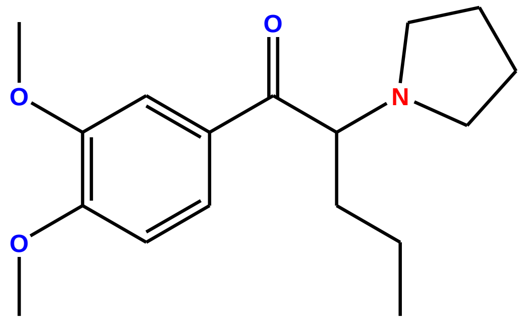	CCCC(C(=O)C1=CC(=C(C=C1)OC)OC)N2CCCC2	38.8
**4-Bromo-α-pyrrolidinopentiophenone** (1-(4-Bromophenyl)-2-(pyrrolidin-1-yl)pentan-1-one)	4-BrPVP	850352-59-9	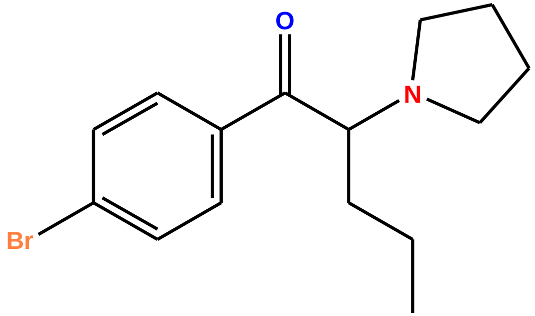	CCCC(C(=O)C1=CC=C(C=C1)Br)N2CCCC2	20.3
**4-Fluoro-α-pyrrolidinohexanophenone** (1-(4-Fluorophenyl)-2-(pyrrolidin-1-yl)hexan-1-one)	4-FPHP	2230706-09-7	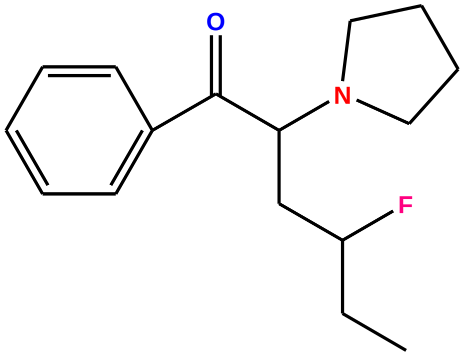	CCCCC(C(=O)C1=CC=C(C=C1)F)N2CCCC2	20.3

**4-Methoxy-α-pyrrolidinoheptanophenone** (1-(4-Methoxyphenyl)-2-(pyrrolidine-1-yl)heptan-1-one)	4-MeO-α-PHPP	1801552-04-4	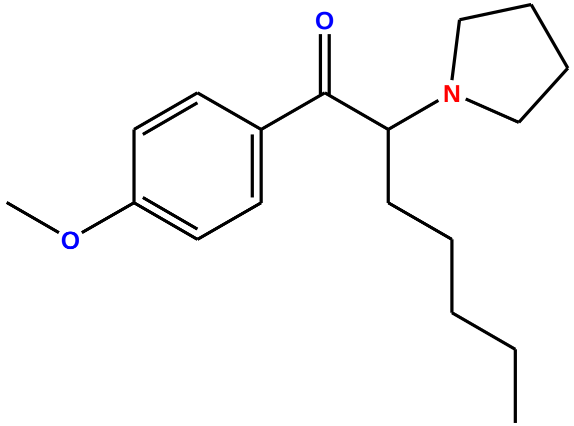	CCCCCC(C(=O)C1=CC=C(C=C1)OC)N2CCCC2	29.5
**4-Methoxy-α-pyrrolidinooctanophenone** (1-(4-Methoxyphenyl)-2-(pyrrolidine-1-yl)octan-1-one)	4-MeO-α-POP	1800098-37-6	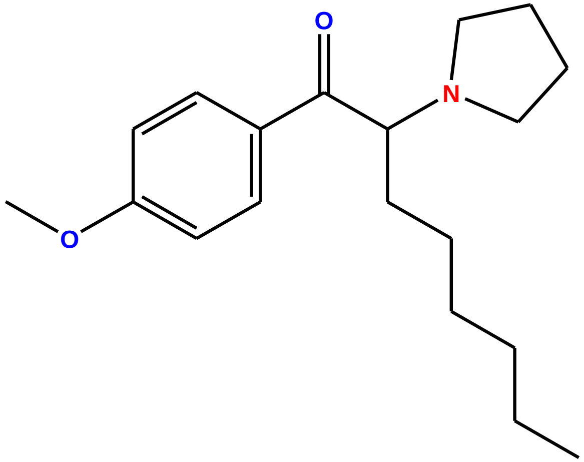	CCCCCCC(C(=O)C1=CC=C(C=C1)OC)N2CCCC2	29.5

**4-Methoxy-α-pyrrolidinovalerophenone** (1-(4-Methoxyphenyl)-2-(pyrrolidin-1-yl)pentan-1-one)	4-MeO-α-PVP	14979-97-6	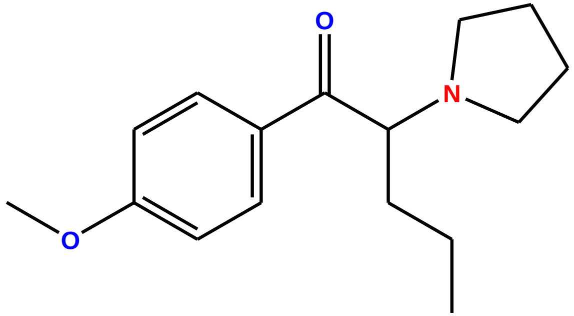	CCCC(C(=O)C1=CC=C(C=C1)OC)N2CCCC2	29.5
**2-(Methylamino)-1-(naphthalen-2-yl)propan-1-one**	BMAPN	109453-73-8	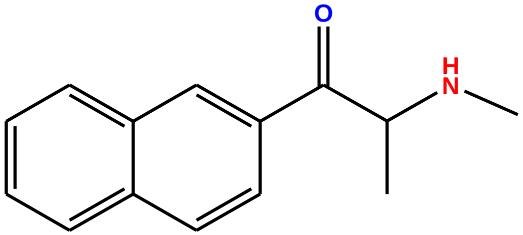	CC(C(=O)C1=CC2=CC=CC=C2C=C1)NC	29.1
**Dimethylpentedrone** (2-(Dimethylamino)-1-(phenyl)pentan-1-one)	DMP	2168229-67-0	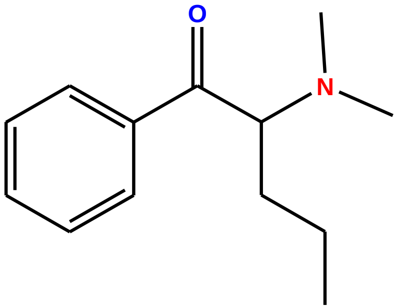	CCCC(C(=O)C1=CC=CC=C1)N(C)C	20.3

**α-Propyloaminopentiophenone or *N*-propylpentedrone** (1-(Phenyl)-2-(propylamino)pentan-1-one)	α-PPP	747345-91-1	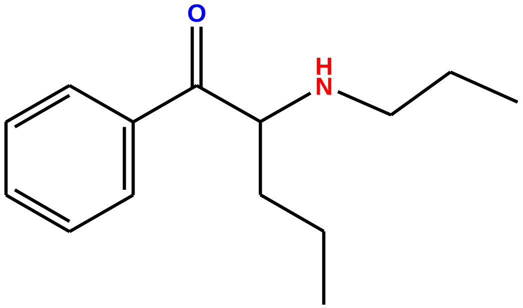	CCCC(C(=O)C1=CC=CC=C1)NC CC	29.1
**α-Pyrrolidinohexanophenone** (1-(Phenyl)-2-(1-pyrrolidinyl)hexan-1-one)	α-PHP	13415-86-6	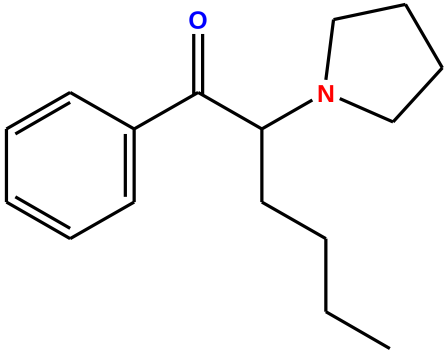	CCCCC(C(=O)C1=CC=CC=C1)N 2CCCC2	20.3
**α-Pyrrolidinoisohexanophenone** (4-(Methyl)-1-(phenyl)-2-(pyrrolidin-1-yl)pentan-1-one)	α-PIHP	2181620-71-1	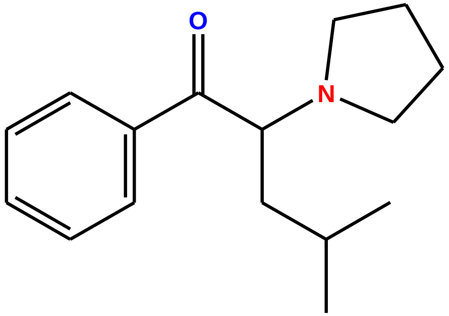	CC(C)CC(C(=O)C1=CC=CC=C1) N2CCCC2	20.3

**Pentedrone** (2-(Methylamino)-1-(phenyl)pentan-1-one)	PTD	879722-57-3	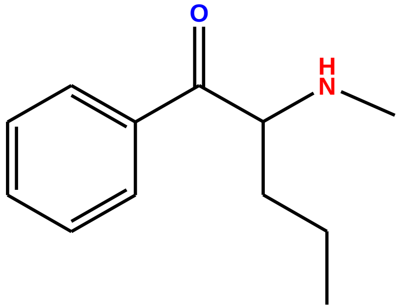	CCCC(C(=O)C1=CC=CC=C1)NC	29.1

α-BHP: α-butylaminohexanophenone; **bk-EBDB:** eutylone; **bk-EBDP:** *N*-ethylpentylone; **bk-MAP:** methylone; **bk-MBDP:** pentylone; **bk-MDEA:** ethylone; **BMAPN:** 2-(methylamino)-1-(naphthalen-2-yl)propan-1-one; **4-BMC:** 4-bromomethcathinone or brephedrone; **BPD:** buphedrone; **4-BrPVP:** 4-bromo-α-pyrrolidinopentiophenone; **BTL:** butylone; **CAS:** Chemical Abstracts Service; **CATH:** cathinone or norephedrone; **2,4-DMEC:** 2,4-dimethylethcathinone; **2,4-DMMC:** 2,4-dimethylmethcathinone or 2-methylmephedrone; **3,4-DMMC:** 3,4-dimethylmethcathinone; **DMP:** dimethylpentedrone; **3,4-DMPVP:** 3,4-dimethoxy-α-pyrrolidinopentiophenone; **EPH:** methcathinone or ephedrone; **EPP:** ethcathinone; **3-FMC:** 3-fluoromethcathinone or 3-flephedrone; **4-FMC:** 4-fluoromethcathinone or flephedrone; **4-FPHP:** 4-fluoro-α-pyrrolidinohexanophenone; **IUPAC:** International Union of Pure and Applied Chemistry; **MDPBP:** 3,4-methylenedioxy-α-pyrrolidinobutyrophenone; **MDPV:** 3,4-methylenedioxypyrovalerone; **4-MEC:** 4-methylethcathinone; **4-MeO-α-PHPP:** 4-methoxy-α-pyrrolidinoheptanophenone; **4-MeO-α-POP:** 4-methoxy-α-pyrrolidinooctanophenone; **4-MeO-α-PVP:** 4-methoxy-α-pyrrolidinovalerophenone; **3-MMC:** 3-methylmethcathinone; **4-MMC:** mephedrone or 4-methylmethcathinone; **4-MPBP:** 4-methyl-α-pyrrolizinobutyrophenone; **4-MPD:** 4-methylpentedrone; **MPH:** hexedrone; **MPP:** pyrovalerone; **MTP:** thiothinone; **NPP:** naphthylpyrovalerone or naphyrone; **α-PHP:** α-pyrrolidinohexanophenone; **α-PIHP:** α-pyrrolidinoisohexanophenone; **5-PPDI:** indanyl-α-pyrrolidinobutiophenone; **α-PPP:** α-propyloaminopentiophenone or *N*-propylpentedrone; **PSA:** polar surface area; **PTD:** pentedrone; **α-PVP:** α-pyrrolidinovalerophenone; **SC:** synthetic cathinones; **SMILES:** Simplified Molecular Input Line Entry System; **(*S*)-MTFP:** (*S*)-metamfepramone or *N*,*N*-dimethylcathinone; **TBCP:** bupropion or amfebutamone.

Table S2: Brief summary of predicted data (physical-chemical properties (i.e., WSol and log K_{OW}) and toxicity) for 44 SC using EPI Suite™ program (green algae, daphnid and fish) and TEST™ program (protozoan, *Tetrahymena pyriformis*).

Software		Parameter		SC order effects
EPI Suite™ program	KOWWIN™	Log K_{OW}		• Log K_{OW} < 3.00: CATH (1.38) < MTP (1.67) < EPH (1.85) < bk-MAP (1.91) < 4-FMC and 3-FMC (2.05) < (*S*)-MTFP (2.06) < BPD and EPP (2.34) < 3-MMC and 4-MMC (2.39) < BTL and bk-MDEA (2.40) < 4-BMC (2.74) < PTD (2.83) < bk-EBDB, bk-MBDP and 4-MEC (2.89) < 3,4-DMMC and 2,4-DMMC (2.94).<br>• Log K_{OW} > 3.00: BMAPN (3.02) < DMP (3.04) < MPH (3.32) < bk-EBDP and 4-MPD (3.38) < 2,4-DMEC (3.43) < MDPBP (3.48) < 3,4-DMPVP (3.56) < α-PPP (3.81) < TBCP (3.85) < α-PVP (3.91) < MDPV and 4-MPBP (3.97) < 4-MeO-α-PVP (3.99) < α-PIHP (4.33) < α-PHP (4.40) < MPP (4.46) < 4-FPHP (4.60) < α- BHP (4.79) < 4-BrPVP (4.80) < 5-PPDI (4.89) < 4-MeO-α-PHPP (4.97) < NPP (5.09) < 4-MeO-α-POP (5.47).
	WSKOWWIN™	**WSol** ($mg\ L^{-1}$ at 25 °C)		• 4-MeO-α-POP (2.57) < NPP (7.25) < 4-MeO-α-PHPP (8.14) < 4-BrPVP (8.65) < 5-PPDI (14.53) < α-BHP (20.05) < 4-FPHP (23.81) < MPP (39.83) < α-PHP (44.49) < α-PIHP (51.40) < MDPV (70.24) < 4-MeO-α-PVP (81.19) < 4-MPBP (124.80) < 3,4-DMPVP (128.90) < α-PVP (139.40) < TBCP (140.20) < α-PPP (196.30) < MDPBP (221.50) < bk-EBDP (313.90) < 2,4-DMEC (489.40) < 4-MPD (546.60) < MPH (610.50) < bk-EBDB and bk-MBDP (984.30) < BMAPN (995.50) < DMP (1059.00) < 4-BMC (1223.00) < 3,4-DMMC and 2,4-DMMC (1515.00) < 4-MEC (1692.00) < PTD (1890.00) < BTL and bk-MDEA (3076.00) < 3-MMC and 4-MMC (5211.00) < BPD and EPP (5819.00) < bk-MAP (9571.00) < 4-FMC and 3-FMC (9860.00) < (*S*)-MTFP (10090.00) < EPH (17810.00) < MTP (23770.00) < CATH (51470.00).
	ECOSAR™	Predicted acute toxicity ($mg\ L^{-1}$)	Fish (96 h/LC_{50})	• 4-MeO-α-POP (0.308) < NPP (0.507) < 4-MeO-α-PHPP (0.618) < 5-PPDI (0.621) < α-BHP (0.693) < 4-BrPVP (0.861) < 4-FPHP (0.987) < MPP (1.144) < α-PHP (1.245) < α-PIHP (1.392) < 4-MPBP (2.267) < 4-MeO-α-PVP (2.467) < α-PVP (2.468) < MDPV (2.675) < α-PPP (2.717) < TBCP (2.786) < 2,4-DMEC (4.510) < 4-MPD (4.910) < 3,4-DMPVP (5.318) < MDPBP (5.336) < MPH (5.346) < bk-EBDP (5.911) < DMP (8.164) < BMAPN (8.707) < 3,4-DMMC and 2,4-DMMC (8.832) < 4-MEC (9.616) < PTD (10.469) < bk-EBDB and bk-MBDP (11.727) < 4-BMC (15.235) < 3-MMC and 4-MMC (18.731) < BPD and EPP (20.393) < BTL and bk-MDEA (23.181) < (*S*)-MTFP (31.145) < 4-FMC and 3-FMC (32.366) < EPH (39.476) < bk-MAP (45.640) < MTP (53.728) < CATH (73.014).
			Daphnid (48 h/LC_{50})	• 4-MeO-α-POP (0.055) < NPP (0.086) < 5-PPDI and 4-MeO-α-PHPP (0.104) < α-BHP (0.114) < 4-BrPVP (0.142) < 4-FPHP (0.159) < MPP (0.181) < α-PHP (0.196) < α-PIHP (0.217) < 4-MPBP (0.340) < α-PVP (0.367) < 4-MeO-α-PVP (0.371) < α-PPP (0.400) < MDPV (0.401) < TBCP (0.412) < 2,4-DMEC (0.635) < 4-MPD (0.687) < MPH (0.743) < MDPBP (0.756) < 3,4-DMPVP (0.760) < bk-EBDP (0.828) < DMP (1.098) < BMAPN (1.169) < 3,4-DMMC and 2,4-DMMC (1.175) < 4-MEC (1.271) < PTD (1.374) < bk-EBDB and bk-MBDP (1.551) < 4-BMC (1.979) < 3-MMC and 4-MMC (2.338) < BPD and EPP (2.529) < BTL and bk-MDEA (2.895) < (*S*)-MTFP (3.738) < 4-FMC and 3-FMC (3.880) < EPH (4.623) < bk-MAP (5.384) < MTP (6.162) < CATH (8.100).

			**Green algae** **(96 h/EC_{50})**	• 4-MeO-α-POP (0.020) < NPP (0.035) < 4-MeO-α-PHPP (0.043) < 5-PPDI (0.044) < α-BHP (0.050) < 4-BrPVP (0.062) < 4-FPHP (0.072) < MPP (0.085) < α-PHP (0.093) < α-PIHP (0.105) < 4-MPBP (0.178) < 4-MeO-α-PVP (0.193) < α-PVP (0.195) < MDPV (0.210) < α-PPP (0.217) < TBCP (0.222) < 2,4-DMEC (0.377) < 4-MPD (0.413) < 3,4-DMPVP (0.438) < MDPBP (0.443) < MPH (0.452) < bk-EBDP (0.497) < DMP (0.713) < BMAPN (0.762) < 3,4-DMMC and 2,4-DMMC (0.780) < 4-MEC (0.855) < PTD (0.937) < bk-EBDB and bk-MBDP (1.042) < 4-BMC (1.378) < 3-MMC and 4-MMC (1.761) < BPD and EPP (1.930) < BTL and bk-MDEA (2.178) < (*S*)-MTFP (3.043) < 4-FMC and 3-FMC (3.166) < EPH (3.951) < bk-MAP (4.535) < MTP (5.488) < CATH (7.705).
		**Predicted chronic toxicity (ChV)**	**Fish** **($mg\ L^{-1}$)**	• 4-MeO-α-POP (0.005) < NPP (0.010) < 5-PPDI and 4-MeO-α-PHPP (0.013) < α-BHP (0.015) < 4-BrPVP (0.019) < 4-FPHP (0.023) < MPP (0.028) < α-PHP (0.031) < α-PIHP (0.036) < 4-MPBP (0.066) < 4-MeO-α-PVP (0.071) < α-PVP (0.073) < MDPV (0.077) < α-PPP (0.083) < TBCP (0.084) < 2,4-DMEC (0.158) < 4-MPD (0.175) < 3,4-DMPVP (0.178) < MDPBP (0.184) < MPH (0.195) < bk-EBDP (0.211) < DMP (0.329) < BMAPN (0.353) < 3,4-DMMC and 2,4-DMMC (0.368) < 4-MEC (0.409) < PTD (0.454) < bk-EBDB and bk-MBDP (0.498) < 4-BMC (0.683) < 3-MMC and 4-MMC (0.948) < BPD and EPP (1.053) < BTL and bk-MDEA (1.171) < (*S*)-MTFP (1.776) < 4-FMC and 3-FMC (1.853) < EPH (2.426) < bk-MAP (2.744) < MTP (3.519) < CATH (5.292).
			**Daphnid** **($mg\ L^{-1}$)**	• 4-MeO-α-POP (0.007) < NPP (0.010) < 5-PPDI and 4-MeO-α-PHPP (0.012) < α-BHP (0.013) < 4-BrPVP (0.016) < 4-FPHP (0.018) < MPP (0.020) < α-PHP (0.021) < α-PIHP (0.023) < 4-MPBP (0.035) < α-PVP (0.037) < 4-MeO-α-PVP (0.038) < α-PPP (0.040) < MDPV (0.041) < TBCP (0.042) < 2,4-DMEC (0.061) < 4-MPD (0.066) < MPH (0.071) < MDPBP (0.073) < 3,4-DMPVP (0.074) < bk-EBDP (0.079) < DMP (0.102) < 3,4-DMMC and 2,4-DMMC (0.107) < BMAPN (0.108) < 4-MEC (0.115) < PTD (0.124) < bk-EBDB and bk-MBDP (0.141) < 4-BMC (0.177) < 3-MMC and 4-MMC (0.201) < BPD and EPP (0.216) < BTL and bk-MDEA (0.249) < (*S*)-MTFP (0.309) < 4-FMC and 3-FMC (0.320) < EPH (0.373) < bk-MAP (0.438) < MTP (0.488) < CATH (0.620).
			**Green algae** **($mg\ L^{-1}$)**	• 4-MeO-α-POP (0.009) < NPP (0.015) < 4-MeO-α-PHPP (0.018) < 5-PPDI (0.019) < α-BHP (0.021) < 4-BrPVP (0.026) < 4-FPHP (0.030) < MPP (0.035) < α-PHP (0.038) < α-PIHP (0.042) < 4-MPBP (0.070) < α-PVP and 4-MeO-α-PVP (0.076) < MDPV (0.082) < α-PPP (0.084) < TBCP (0.086) < 2,4-DMEC (0.141) < 4-MPD (0.154) < 3,4-DMPVP (0.166) < MDPBP (0.167) < MPH (0.168) < bk-EBDP (0.185) < DMP (0.258) < BMAPN (0.276) < 3,4-DMMC and 2,4-DMMC (0.280) < 4-MEC (0.306) < PTD (0.333) < bk-EBDB and bk-MBDP (0.373) < 4-BMC (0.487) < 3-MMC and 4-MMC (0.604) < BPD and EPP (0.659) < BTL and bk-MDEA (0.748) < (*S*)-MTFP (1.015) < 4-FMC and 3-FMC (1.055) < EPH (1.295) < bk-MAP (1.494) < MTP (1.772) < CATH (2.429).

	**BCFBAF™**	**Estimated BCF[1] (L Kg^{-1} wet wt^{-1})**	**Fish UT**	• < 2000.000 L Kg^{-1} wet wt^{-1} (Not bioaccumulative): CATH (2.947) < MTP (5.364) < (*S*)-MTFP (5.591) < EPH (7.413) < bk-MAP (9.078) < 4-FMC and 3-FMC (12.320) < BPD and EPP (20.170) < DMP (21.390) < 3-MMC and 4-MMC (25.300) < BTL and bk-MDEA (25.650) < α-PVP (32.920) < 5-PPDI (42.730) < 3,4-DMPVP (42.960) < α-PIHP (44.290) < α-PHP (49.750) < PTD (53.500) < MDPBP (54.280) < 4-BMC (54.330) < 4-MEC (71.780) < bk-EBDB and bk-MBDP (72.840) < 3,4-DMMC and 2,4-DMMC (74.450) < 4-MPBP (81.370) < MDPV (83.320) < 4-MeO-α-PVP (88.810) < BMAPN (104.300) < MPP (117.200) < MPH (131.600) < 2,4-DMEC (185.100) < 4-MPD (191.700) < 4-MeO-α-PHPP (191.800) < bk-EBDP (194.700) < 4-FPHP (201.000) < 4-BrPVP (205.000) < 4-MeO-α-POP (255.800) < α-PPP (259.000) < NPP (373.800) < TBCP (573.300) < α-BHP (793.200).
			**Fish MT**	• < 2000.000 L Kg^{-1} wet wt^{-1} (Not bioaccumulative): CATH (2.407) < MTP (3.959) < (*S*)-MTFP (5.416) < EPH (5.429) < bk-MAP (6.339) < 4-FMC and 3-FMC (8.423) < BPD and EPP (14.540) < 3-MMC and 4-MMC (17.270) < BTL and bk-MDEA (17.490) < DMP (24.400) < 4-BMC (36.800) < PTD (40.350) < α-PVP (43.170) < 4-MEC (49.950) < bk-EBDB and bk-MBDP (50.650) < 3,4-DMPVP (52.300) < 3,4-DMMC and 2,4-DMMC (54.140) < 5-PPDI (58.090) < α-PIHP (59.290) < MDPBP (62.650) < α-PHP (66.710) < BMAPN (70.280) < 4-MPBP (102.100) < MDPV (104.500) < MPH (108.600) < 4-MeO-α-PVP (111.200) < 4-MPD (142.100) < 2,4-DMEC (147.500) < MPP (154.200) < bk-EBDP (144.200) < α-PPP (249.700) < 4-MeO-α-PHPP (257.900) < 4-FPHP (262.000) < 4-BrPVP (272.200) < 4-MeO-α-POP (348.200) < TBCP (424.800) < NPP (497.600) < α-BHP (961.100).
			**Fish LT**	• < 2000.000 L Kg^{-1} wet wt^{-1} (Not bioaccumulative): CATH (2.257) < MTP (3.612) < (*S*)-MTFP (5.239) < EPH (4.923) < bk-MAP (5.689) < 4-FMC and 3-FMC (7.509) < BPD and EPP (13.040) < 3-MMC and 4-MMC (15.320) < BTL and bk-MDEA (15.520) < DMP (24.860) < 4-BMC (32.530) < PTD (36.450) < 4-MEC (44.370) < bk-EBDB and bk-MBDP (44.970) < α-PVP (46.600) < 3,4-DMMC and 2,4-DMMC (48.510) < 3,4-DMPVP (54.630) < BMAPN (62.010) < 5-PPDI (63.790) < MDPBP (64.060) < α-PIHP (64.610) < α-PHP (72.740) < MPH (100.100) < 4-MPBP (108.000) < MDPV (110.500) < 4-MeO-α-PVP (117.500) < 4-MPD (127.800) < bk-EBDP (129.600) < 2,4-DMEC (134.800) < MPP (166.500) < α-PPP (240.100) < 4-MeO-α-PHPP (281.300) < 4-FPHP (281.800) < 4-BrPVP (295.200) < TBCP (381.700) < 4-MeO-α-POP (382.100) < NPP (540.200) < α-BHP (1001.000).
		**Estimated BAF[1] (L Kg^{-1} wet wt^{-1})**	**Fish UT**	• < 2000.000 L Kg^{-1} wet wt^{-1} (Not bioaccumulative): CATH (2.947) < MTP (5.364) < (*S*)-MTFP (5.591) < EPH (7.413) < bk-MAP (9.078) < 4-FMC and 3-FMC (12.320) < BPD and EPP (20.170) < DMP (21.390) < 3-MMC and 4-MMC (25.300) < BTL and bk-MDEA (25.660) < α-PVP (32.920) < 5-PPDI (42.730) < 3,4-DMPVP (42.960) < α-PIHP (44.290) < α-PHP (49.750) < PTD (53.500) < MDPBP (54.280) < 4-BMC (54.330) < 4-MEC (71.790) < bk-EBDB and bk-MBDP (72.850) < 3,4-DMMC and 2,4-DMMC (74.460) < 4-MPBP (81.370) < MDPV (83.320) < 4-MeO-α-PVP (88.820) < BMAPN (104.300) < MPP (117.200) < MPH (131.600) < 2,4-DMEC (185.200) < 4-MPD (191.700) < 4-MeO-α-PHPP (192.000) < bk-EBDP (194.800) < 4-FPHP (201.100) < 4-BrPVP (205.200) < 4-MeO-α-POP (257.400) < α-PPP (259.100) < NPP (375.900) < TBCP (579.600) < α-BHP (805.400).

			**Fish MT**	• < 2000.000 L Kg^{-1} wet wt^{-1} (Not bioaccumulative): CATH (2.407) < MTP (3.960) < (*S*)-MTFP (5.416) < EPH (5.429) < bk-MAP (6.340) < 4-FMC and 3-FMC (8.424) < BPD and EPP (14.540) < 3-MMC and 4-MMC (17.270) < BTL and bk-MDEA (17.500) < DMP (24.400) < 4-BMC (36.830) < α-PVP (43.190) < PTD (49.360) < 4-MEC (49.990) < bk-EBDB and bk-MBDP (50.690) < 3,4-DMPVP (52.320) < 3,4-DMMC and 2,4-DMMC (54.170) < 5-PPDI (58.400) < α-PIHP (59.380) < MDPBP (62.670) < α-PHP (66.840) < BMAPN (70.450) < 4-MPBP (102.300) < MDPV (104.600) < MPH (108.700) < 4-MeO-α-PVP (111.400) < 4-MPD (142.500) < 2,4-DMEC (147.900) < MPP (155.000) < bk-EBDP (144.600) < α-PPP (250.800) < 4-FPHP and 4-MeO-α-PHPP (265.500) < 4-BrPVP (278.000) < 4-MeO-α-POP (391.900) < TBCP (435.800) < NPP (535.500) < α-BHP (1047.000).
			**Fish LT**	• < 2000.000 L Kg^{-1} wet wt^{-1} (Not bioaccumulative): CATH (2.257) < MTP (3.612) < (*S*)-MTFP (5.239) < EPH (4.924) < bk-MAP (5.691) < 4-FMC and 3-FMC (7.514) < BPD and EPP (13.050) < 3-MMC and 4-MMC (15.340) < BTL and bk-MDEA (15.530) < DMP (24.880) < 4-BMC (32.610) < PTD (36.510) < 4-MEC (44.490) < bk-EBDB and bk-MBDP (45.100) < α-PVP (46.910) < 3,4-DMMC and 2,4-DMMC (48.630) < 3,4-DMPVP (54.830) < BMAPN (62.370) < MDPBP (64.290) < α-PIHP (65.910) < 5-PPDI (68.350) < α-PHP (74.570) < MPH (100.600) < 4-MPBP (109.400) < MDPV (111.900) < 4-MeO-α-PVP (119.100) < 4-MPD (129.000) < bk-EBDP (130.900) < 2,4-DMEC (136.000) < MPP (174.000) < α-PPP (244.600) < 4-FPHP (305.000) < 4-BrPVP (333.600) < 4-MeO-α-PHPP (333.900) < TBCP (399.700) < 4-MeO-α-POP (640.200) < NPP (724.200) < α-BHP (1263.000).
		**Estimated BCF**[2] **(L Kg^{-1} wet wt^{-1})**	**Fish UT**	• < 2000.000 L Kg^{-1} wet wt^{-1} (Not bioaccumulative): CATH (3.469) < MTP (5.871) < EPH (8.424) < bk-MAP (9.577) < 4-FMC and 3-FMC (12.840) < (*S*)-MTFP (13.140) < BPD and EPP (24.210) < 3-MMC and 4-MMC (27.430) < BTL and bk-MDEA (27.780) < 4-BMC (59.250) < PTD (73.020) < 4-MEC (82.960) < bk-EBDB and bk-MBDP (84.040) < 3,4-DMMC and 2,4-DMMC (94.260) < BMAPN (113.400) < DMP (118.000) < MPH (223.300) < 4-MPD (253.700) < bk-EBDP (257.000) < 2,4-DMEC (288.300) < MDPBP (321.800) < 3,4-DMPVP (380.000) < α-PPP (679.400) < TBCP (747.100) < α-PVP (847.900) < 4-MPBP (961.200) < MDPV (973.500) < 4-MeO-α-PVP (1016.000).<br>• 2000.000 ≤ 5000.000 L Kg^{-1} wet wt^{-1} (Bioaccumulative): α-PIHP (2121.000) < α-PHP (2480.000) < MPP (2790.000) < 4-FPHP (3752.000).<br>• > 5000.000 L Kg^{-1} wet wt^{-1} (Very bioaccumulative): α-BHP (5456.000) < 4-BrPVP (5524.000) < 5-PPDI (6543.000) < 4-MeO-α-PHPP (7541.000) < NPP (9086.000) < 4-MeO-α-POP (15020.000).
		**Estimated BAF**[2] **(L Kg^{-1} wet wt^{-1})**		• < 2000.000 L Kg^{-1} wet wt^{-1} (Not bioaccumulative): CATH (3.510) < MTP (5.964) < EPH (8.587) < bk-MAP (9.777) < 4-FMC and 3-FMC (13.160) < (*S*)-MTFP (13.470) < BPD and EPP (25.150) < 3-MMC and 4-MMC (28.610) < BTL and bk-MDEA (28.980) < 4-BMC (64.050) < PTD (80.130) < 4-MEC (92.020) < bk-EBDB and bk-MBDP (93.330) < 3,4-DMMC and 2,4-DMMC (105.800) < BMAPN (130.000) < DMP (135.900) < MPH (285.200) < 4-MPD (333.500) < bk-EBDP (338.900) < 2,4-DMEC (391.000) < MDPBP (449.500) < 3,4-DMPVP (559.200) < α-PPP (1247.000) < TBCP (1435.000) < α-PVP (1735.000).<br>• 2000.000 ≤ 5000.000 L Kg^{-1} wet wt^{-1} (Bioaccumulative): 4-MPBP (2104.000) < MDPV (2146.000) < 4-MeO-α-PVP (2293.000).<br>• > 5000.000 L Kg^{-1} wet wt^{-1} (Very bioaccumulative): α-PIHP (7884.000) < α-PHP (10450.000) < MPP (13000.000) < 4-FPHP (22860.000) < α-BHP (48620.000) < 4-BrPVP (49900.000) < 5-PPDI (71490.000) < 4-MeO-α-PHPP (97660.000) < NPP (149800.000) < 4-MeO-α-POP (568100.000).

**TEST™ program**	**Predicted 48 h IGC_{50} (mg L^{-1})**	**Protozoan** *(Tetrahymena pyriformis)*	• NPP (1.11) < 4-MeO-*α*-POP (1.27) < 4-FPHP (1.67) < 4-MeO-*α*-PHPP (2.01) < 4-BrPVP (2.23) < *α*-BHP (2.36) < *α*-PHP (4.25) < MPP (4.33) < TBCP (4.61) < 4-MeO-*α*-PVP (4.85) < *α*-PIHP (5.29) < 5-PPDI (5.30) < *α*-PPP (5.61) < BMAPN (5.85) < 3,4-DMPVP (6.16) < MDPV (6.50) < *α*-PVP (6.54) < 4-MPBP (6.55) < MPH (7.42) < 4-MPD (7.69) < 2,4-DMEC (9.64) < MDPBP (13.42) < 4-BMC (13.81) < 4-MEC (16.91) < 2,4-DMMC (17.09) < bk-EBDP (17.68) < 3,4-DMMC (17.83) < PTD (19.28) < bk-MBDP (25.78) < DMP (26.52) < bk-EBDB (27.48) < EPP (28.95) < MTP (31.57) < 4-MMC (34.76) < 3-MMC (39.03) < BPD (47.47) < bk-MDEA (51.56) < BTL (51.80) < 4-FMC (54.94) < 3-FMC (59.07) < bk-MAP (74.66) < (*S*)-MTFP (94.97) < EPH (97.31) < CATH (148.21).

BAF: bioaccumulation factor; **BCF:** bioconcentration factor; ***α*-BHP:** *α*-butylaminohexanophenone; **bk-EBDB:** eutylone; **bk-EBDP:** *N*-ethylpentylone; **bk-MAP:** methylone; **bk-MBDP:** pentylone; **bk-MDEA:** ethylone; **BMAPN:** 2-(methylamino)-1-(naphthalen-2-yl)propan-1-one; **4-BMC:** 4-bromomethcathinone or brephedrone; **BPD:** buphedrone; **4-BrPVP:** 4-bromo-*α*-pyrrolidinopentiophenone; **BTL:** butylone; **CATH:** cathinone or norephedrone; **ChV:** chronic effects values; **2,4-DMEC:** 2,4-dimethylethcathinone; **2,4-DMMC:** 2,4-dimethylmethcathinone or 2-methylmephedrone; **3,4-DMMC:** 3,4-dimethylmethcathinone; **DMP:** dimethylpentedrone; **3,4-DMPVP:** 3,4-dimethoxy-*α*-pyrrolidinopentiophenone; **EC_{50}:** half maximal effective concentration; **EPH:** methcathinone or ephedrone; **EPP:** ethcathinone; **3-FMC:** 3-fluoromethcathinone or 3-flephedrone; **4-FMC:** 4-fluoromethcathinone or flephedrone; **4-FPHP:** 4-fluoro-*α*-pyrrolidinohexanophenone; **h:** hours; **IGC_{50}:** 50 % of the inhibition growth concentration; **LC_{50}:** half maximal lethal concentration; **Log K_{OW}:** values calculated using the log octanol-water partition coefficient calculation program KOWWIN™; **LT:** lower trophic; **MDPBP:** 3,4-methylenedioxy-*α*-pyrrolidinobutyrophenone; **MDPV:** 3,4-methylenedioxypyrovalerone; **4-MEC:** 4-methylethcathinone; **4-MeO-*α*-PHPP:** 4-methoxy-*α*-pyrrolidinoheptanophenone; **4-MeO-*α*-POP:** 4-methoxy-*α*-pyrrolidinooctanophenone; **4-MeO-*α*-PVP:** 4-methoxy-*α*-pyrrolidinovalerophenone; **3-MMC:** 3-methylmethcathinone; **4-MMC:** mephedrone or 4-methylmethcathinone; **4-MPBP:** 4-methyl-*α*-pyrrolizinobutyrophenone; **4-MPD:** 4-methylpentedrone; **MPH:** hexedrone; **MPP:** pyrovalerone; **MT:** mid trophic; **MTP:** thiothinone; **NPP:** naphthylpyrovalerone or naphyrone; ***α*-PHP:** *α*-pyrrolidinohexanophenone; ***α*-PIHP:** *α*-pyrrolidinoisohexanophenone; **5-PPDI:** indanyl-*α*-pyrrolidinobutiophenone; ***α*-PPP:** *α*-propyloaminopentiophenone or *N*-propylpentedrone; **PTD:** pentedrone; ***α*-PVP:** *α*-pyrrolidinovalerophenone; **SC:** synthetic cathinones; **(*S*)-MTFP:** (*S*)-metamfepramone or *N*,*N*-dimethylcathinone; **TBCP:** bupropion or amfebutamone; **UT:** upper trophic; **WSol:** water solubility; [1]***Arnot-Gobas* method nº 1:** considering biotransformation rate estimates; [2]***Arnot-Gobas* method nº 2:** assuming a biotransformation rate of zero.

1.2. In vivo assays

1.2.1. Sub-chronic assays in *Tetrahymena thermophila*

Sub-chronic ecotoxicity assays were performed on *T. thermophila* for 28 hours (h), and a reference test with $K_2Cr_2O_7$ at five concentrations ranging between 5.60 and 56.00 mg L^{-1} was conducted. The respective results are described in **Table S3**.

Table S3: Percentage of growth inhibition vs log concentration of *Tetrahymena thermophila* after 28 h of exposure to $K_2Cr_2O_7$ at five concentrations tested.

Reference test/Log concentration	Growth inhibition average (%)				
	**0.748**	**1.000**	**1.255**	**1.505**	**1.748**
$K_2Cr_2O_7$	-3	5	50	86	85

2. MATERIALS AND METHODS

2.1. In vivo assays

2.1.1. Sub-chronic assays with *T. thermophila*

A ciliate inoculum was prepared from a stock culture by adjusting optical density (OD) at wavelength (λ) of 440 nm to 0.040 with standard freshwater (SFW) medium. To perform the assays, disposable 1 cm spectrophotometric cells were used to measure the OD. Each test cell contained 40 µL of *T. thermophila* ciliate inoculum and food suspension. Control and five tested synthetic cathinones (SC) were incubated in an Infors HT Ecotron incubator (Fisher Scientific, Portugal) at 30 °C in darkness, for 24 and 28 h. Assays were performed in triplicate and OD measurements at 0, 24 and 28 h were performance using an UV/Vis spectrophotometer (ATI Unicam, Leeds, England) at 440 nm. The OD in the controls after 24 or 28 h of incubation must show a decrease of the T0 value by at least 60 % as the uninhibited growth of the ciliates will consume the food substrate. The percentage of growth inhibition can be calculated from the difference in OD between the control cells and the test cells after 24 or 28 h using the following equation:

$$\%\, Growth\ Inhibition_{(C1-C5)} = \left(1 - \frac{\Delta OD_{(C1-C5)}}{\Delta OD_{(C0)}}\right) \times 100 \qquad (1)$$

where $\Delta OD_{(C1\text{-}C5)}$ is the difference between the absorbance after the incubation (T24h or T28h) and prior to the test (T0h) and $\Delta OD_{(C0)}$ is the absorbance in the control tests.

2.1.2. Sublethal assay with *Daphnia magna*

2.1.2.1. Culture maintenance of microalgae *Raphidocelis subcapitata*

The microalgae *R. subcapitata* culture medium is prepared using the following macronutrients stock solutions: 2.55 g of $NaNO_3$; 1.22 g of $MgCl_2.6H_2O$; 0.44 g of $CaCl_2.2H_2O$; 1.47 g of $MgSO_4.7H_2O$; 0.10 g of K_2HPO_4; and 1.50 g of $NaHCO_3$ prepared in 100 mL of distilled water and stored in an amber glass bottles at 4 °C. A micronutrients stock solution is prepared containing the following substances: 46.38 mg of H_3BO_3, 103.85 mg of $MnCl_2.4H_2O$, 0.82 mg of $ZnCl_2$, 39.94 mg of $FeCl_3.6H_2O$, 0.36 mg of $CoCl_2.6H_2O$, 1.82 mg of $Na_2MoO_4.2H_2O$ and 75.00 mg of $Na_2EDTA.2H_2O$ prepared in 250 mL of distilled water and stored in an amber glass bottle at 4 °C. About 1 mL of each macronutrient stock solution and 1 mL of micronutrients stock solution is added each 1 L of distilled water and pH adjusted to 7.5 ± 0.1.

The culture is maintained for 7 days in the bioterium at a constant temperature (20 ± 2 °C) with illumination at 6000 *lux* and a photoperiod of 16:8 h light: dark.

2.1.2.2. Biochemical determinations

The *Bradford* assay is a colorimetric technique used for protein quantification. A calibration curve with bovine serum albumin (BSA) was performed at a concentration range of 0.0015 to 0.0250 mg mL^{-1}. Each sample (2 µL diluted in 98 µL of phosphate buffer solution (PBS)) were

incubate for 5 min at room temperature (RT) with 100 μL of Bradford reagent. The absorbance was read at λ of 595 nm in a microplate reader and results express as mg mL^{-1} of BSA [1, 2].

Catalase (CAT) activity was determined with a spectrophotometric method at λ of 415 nm and 25 °C. Briefly, the standards of CAT and samples (50 μL) were incubated with 100 μL of 60 μM H_2O_2/60 mM sodium phosphate buffer (SPB) at 37 °C for 1 min. The enzymatic reaction was stopped by the addition of 250 μL of 32 mM ammonium molybdate tetrahydrate (AMT), incubate at RT for 5 min. Blanks were prepared with PBS, 60 μM H_2O_2/60 mM SPB and 32 mM AMT. CAT activity is determined by a calibration curve of CAT (concentration range of 0.156 to 7.000 U mL^{-1}), and results express as U of CAT per mg of protein [3-5].

For Reactive oxygen species (ROS) assay, the 2′,7′-dichlorofluorescin diacetate (H_2DCFDA), a fluorescent probe was used. For that, 10 μL of each sample, 110 μL of PBS and 8.3 μL of 21 mM H_2DCFDA are incubate at 37 °C for 30 min. Fluorescence was read at 25 °C with an excitation λ of 485 nm and an emission λ of 528 nm in a microplate reader. Results was express as μmol of DCF per mg of protein, through a DCF calibration curve (concentration range of 0.078125 to 20.00 μM) [6, 7].

Lipid peroxidation was measured through the thiobarbituric acid reactive substances (TBARS) assays. Samples (10 μL) with ultrapure water (UPW; 70 μL), 50 mM PBS (50 μL), 1 mM BHT (2,6-di-tert-butyl-4-methylphenol; 10 μL), 1.3 % TBA/0.3 % NaOH (75 μL) and 50 % trichloroacetic acid (50 μL) were incubated for 2 h at 60 °C. After cool for 15 min on ice, the final reagent was added, the 20 % sodium dodecyl sulphate (10 μL). Absorbance was measure at λ of 530 nm in a microplate reader and the results express as μmol of MDA per mg of protein, considering the calibration curve of MDA (concentration range of 2.5 to 100.0 μM) [8-10].

A colorimetric assay for detection of acetylcholinesterase (AChE) activity was based on an improved *Ellman* method. For the assay 20 μL of samples was used with 120 μL of 0.5 mM DTNB (diluted in 0.05 M tris buffer) and 60 μL of 20 mM iodinated acetylthiocholine and incubated for 5 min at RT. Absorbance was read in a microplate reader at λ of 412 nm at 25 °C for 3 min [11, 12]. The AChE concentration was calculated following the next formula of extinction coefficient (Ɛ):

$$\varepsilon = \frac{A}{c \times l} \quad (2)$$

where TNB $\varepsilon_{412\ nm}$ of 14.1 × 10^3 M cm^{-1} (14.1 mL $mmol^{-1}$ cm^{-1}), optical path (l) of 0.8 cm, absorbance of sample (A) and molar concentration (c). AChE concentration (mol mL^{-1}) results were expressed as mmol of TNB per mg of protein.

References

1. Ding J.; Zou H.; Liu Q.; Zhang S.; Razanajatovo R. Bioconcentration of the antidepressant fluoxetine and its effects on the physiological and biochemical status in *Daphnia magna*. *Ecotoxicol Environ Saf* **2017**, *142*, 102-109. Doi: 10.1016/j.ecoenv.2017.03.042.
2. Bradford M. A rapid and sensitive method for the quantitation of microgram quantities of protein utilizing the principle of protein-dye binding. *Anal Biochem* **1976**, *72*, 248-254.
3. Bownik A.; Stepniewska Z.; Skowronski T. Protective effects of ectoine on heat-stressed *Daphnia magna*. *J Comp Physiol B* **2014**, *184*(8), 961-976. Doi: 10.1007/s00360-014-0860-x.
4. Bownik A.; Stepniewska Z. Protective effects of ectoine on behavioral, physiological and biochemical parameters of *Daphnia magna* subjected to hydrogen peroxide. *Comp Biochem Physiol C Toxicol Pharmacol* **2015**, *170*, 38-49. Doi: 10.1016/j.cbpc.2015.02.002.
5. Góth L. A simple method for determination of serum catalase activity and revision of reference range *Clin Chim Acta* **1991**, *196* 143-152.
6. De Felice B.; Salgueiro-González N.; Castiglioni S.; Saino N.; Parolini M. Biochemical and behavioral effects induced by cocaine exposure to *Daphnia magna*. *Sci Total Environ* **2019**, *689*, 141-148. Doi: 10.1016/j.scitotenv.2019.06.383.
7. Deng J.; Yu L.; Liu C.; Yu K.; Shi X.; Yeung L.; Lam P.; Wu R.; Zhou B. Hexabromocyclododecane-induced developmental toxicity and apoptosis in zebrafish embryos. *Aquat Toxicol* **2009**, *93*(1), 29-36. Doi: 10.1016/j.aquatox.2009.03.001.

8. Buege J.; Aust S. Microsomal lipid peroxidation. *Microsomal electron transport and CYT P-450* **1978**, *30*, 302-306.
9. Masteling R.; Castro B.; Antunes S.; Nunes B. Whole-organism and biomarker endpoints in *Daphnia magna* show uncoupling of oxidative stress and endocrine disruption in phenolic derivatives. *Ecotoxicol Environ Saf* **2016**, *134*, 64-71. Doi: 10.1016/j.ecoenv.2016.08.012.
10. Ohkawa H.; Ohishi N.; Yagi K. Assay for lipid peroxides in animal tissues by thiobarbituric acid reaction *Anal Biochem* **1979**, *95*, 351-358.
11. Parolini M.; De Felice B.; Ferrario C.; Salgueiro-González N.; Castiglioni S.; Finizio A.; Tremolada P. Benzoylecgonine exposure induced oxidative stress and altered swimming behavior and reproduction in *Daphnia magna*. *Environ Pollut* **2018**, *232*, 236-244. Doi: 10.1016/j.envpol.2017.09.038.
12. Jemec A.; Drobne D.; Tišler T.; Trebše P.; Roš M.; Sepčić K. The applicability of acetylcholinesterase and glutathione *S*-transferase in *Daphnia magna* toxicity test. *Comp Biochem Physiol C Toxicol Pharmacol* **2007**, *144*(4), 303-309. Doi: 10.1016/j.cbpc.2006.10.002.
